# Offshore pelagic subsidies dominate carbon inputs to coral reef predators

**DOI:** 10.1126/sciadv.abf3792

**Published:** 2021-02-19

**Authors:** C. Skinner, A. C. Mill, M. D. Fox, S. P. Newman, Y. Zhu, A. Kuhl, N. V. C. Polunin

**Affiliations:** 1 School of Natural and Environmental Sciences, Newcastle University, Newcastle upon Tyne NE1 7RU, UK.; 2 Department of Ocean Science and Hong Kong Branch of the Southern Marine Science and Engineering, Guangdong Laboratory (Guangzhou), The Hong Kong University of Science and Technology, Kowloon, Hong Kong.; 3 Woods Hole Oceanographic Institution, 266 Woods Hole Rd, Woods Hole, MA 02543, USA.; 4 Banyan Tree Marine Lab, Vabbinfaru Resort, North Malé Atoll, Republic of Maldives.; 5 School of Chemistry, University of Bristol, Cantock’s Close, Bristol BS8 1TS, UK.

## Abstract

Coral reefs were traditionally perceived as productive hot spots in oligotrophic waters. While modern evidence indicates that many coral reef food webs are heavily subsidized by planktonic production, the pathways through which this occurs remain unresolved. We used the analytical power of carbon isotope analysis of essential amino acids to distinguish between alternative carbon pathways supporting four key reef predators across an oceanic atoll. This technique separates benthic versus planktonic inputs, further identifying two distinct planktonic pathways (nearshore reef-associated plankton and offshore pelagic plankton), and revealing that these reef predators are overwhelmingly sustained by offshore pelagic sources rather than by reef sources (including reef-associated plankton). Notably, pelagic reliance did not vary between species or reef habitats, emphasizing that allochthonous energetic subsidies may have system-wide importance. These results help explain how coral reefs maintain exceptional productivity in apparently nutrient-poor tropical settings, but also emphasize their susceptibility to future ocean productivity fluctuations.

## Teaser

Coral reef predators are overwhelmingly sustained by offshore pelagic plankton sources rather than by reef-based sources.

## INTRODUCTION

Ecosystem function relies on the movement and storage of energy and nutrients (*1*). Allochthonous materials can increase local resource availability and affect food web dynamics (*2*), particularly in resource-limited systems. Energetic connectivity is thus a fundamental ecological process for both terrestrial and marine ecosystems. As marine ecosystems are inherently linked by water, their “openness” promotes exchanges of energetic material across their boundaries over multiple spatial scales, and proximity to other habitats facilitates coupling of adjacent food webs (*3*, *4*). Consequently, understanding the trophodynamics (flows of energy) (*5*) and quantifying the primary pathways in marine food webs are challenging.

Traditionally, coral reefs were considered to be productive hot spots in oligotrophic deserts (*6*). However, their food webs are complex, and the mechanisms through which they maintain exceptionally high diversity and biomass are poorly understood. Modern evidence suggests that planktonic production sources are fundamentally important in sustaining coral reef food webs, but our understanding of these reef-pelagic linkages stems from bulk stable isotope data (δ^13^C, δ^15^N, and δ^34^S) (*7*–*10*) and statistical modeling approaches (*11*). Bulk stable isotope data lack resolution; for example, co-occurring sources may not be isotopically distinct (*10*, *12*), thus preventing accurate separation. Isotopic data characterizing food web baselines also vary with environmental conditions (*13*, *14*), requiring robust sampling of dietary sources to compare data across spatial and temporal scales (*15*, *16*). Furthermore, as macromolecules are often not directly routed to consumer tissue, there is a trophic fractionation factor between consumer and diet, which varies substantially among species (*17*). Given these limitations, there is currently no empirical evidence to determine the actual origins of pelagic inputs to coral reefs. However, as climate change is predicted to cause declines in ocean production (*18*), a better understanding of these linkages is crucial.

Compound-specific stable isotope analysis (CSIA) is a new technology that profiles specific biochemical compounds, such as amino acids, and so provides additional resolution for distinguishing the energy sources of consumers. Some amino acids are essential (EAA); consumers cannot synthesize them de novo and must obtain them directly from their diet (*12*). As EAAs are routed to consumer tissues directly, fractionation across trophic levels is minimal, and consumer EAA δ^13^C values (“δ^13^C fingerprints”; δ^13^C_EAA_) (*19*) reflect their baseline dietary carbon sources (*20*). In both terrestrial and aquatic systems, δ^13^C_EAA_ values help distinguish different primary producers based on their biosynthetic pathways of EAA synthesis (*14*, *19*, *21*). Even when bulk stable isotope values vary, primary producer δ^13^C fingerprints are robust to differing growth and environmental conditions (*22*, *23*). Phytoplankton are composed primarily of protein (*24*), and different temporal plankton communities have been distinguished using δ^13^C amino acid values (*22*). This suggests that planktonic sources with different origins may have distinct δ^13^C_EAA_ values, allowing CSIA to trace the origin of the planktonic material subsidizing coral reef food webs.

There is a dynamic and complex mixture of zooplankton available to the reef food web, so we hypothesized several planktonic pathways to reflect this. For example, copepods perpetually dominate reef-associated plankton communities, but species composition and density shifts occur at dusk as nocturnal demersal plankton emerge (*25*). This suggests that diurnal and nocturnal reef-associated plankton carbon pathways may differ. Furthermore, pelagic zooplankton are advected onto the reef from further afield by hydrodynamic processes (e.g., waves or tides) (*26*). They may represent a separate pathway of potentially increased importance in oceanic regions. Previous work has not isotopically separated these pathways, despite the recognized importance of planktonic inputs in supporting many coral reef food webs (*7*–*11*).

**Fig. 1:**
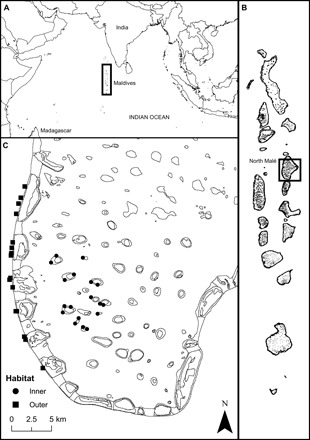
Fish tissue sampling sites around North Malé Atoll, Republic of Maldives. (**A**) Republic of Maldives location in the North Indian Ocean (3.2028°N, 73.2207°E), (**B**) North Malé Atoll in the central Maldives archipelago (4.4167°N, 73.5000°E), and (**C**) sampling sites for fish tissue in the inner lagoonal reefs (●) or along the outer edge reefs (■).

Using δ^13^C_EAA_, we set out (i) to determine whether we could distinguish among the sources of benthic and planktonic inputs that are important in sustaining coral reef food webs and (ii) to quantify their contribution to key reef predators across an atoll seascape. We sampled a range of specialized consumers to represent the different potential planktonic (reef diurnal, reef nocturnal, and pelagic) and benthic reef (algae, coral, and detritus) energy pathways and key fishery target reef predators that occupy the upper trophic level of the food web, across both inner-lagoonal platform reef sites and outer-reef slopes of an oceanic atoll in the Maldives (Fig. 1). The specialized consumer species were chosen as indicators to separate out the distinct carbon pathways that might contribute to the predator food chain (see section S1 for evidence of their carbon source reliance).

We identified several benthic and planktonic carbon pathways available to the reef food web, effectively separating planktonic inputs into two isotopically distinct pathways, nearshore reef-associated plankton and offshore pelagic plankton. Then, using a combination of modeling approaches, namely, δ^13^C fingerprinting of functional pathways (*19*) and Bayesian stable isotope mixing models (*27*), we show that it is predominantly the offshore pelagic plankton pathway that is supporting the predators regardless of reef habitat. Our results provide key insights into the trophodynamics of coral reef ecosystems and their dependence on offshore production, underlining the importance of exogenous inputs to sustaining the productivity of commercially valuable predators in coral reef food webs. Our combined modeling approach is applicable across multiple systems, allowing complex energy fluxes to be better resolved.

## RESULTS

**Fig. 2:**
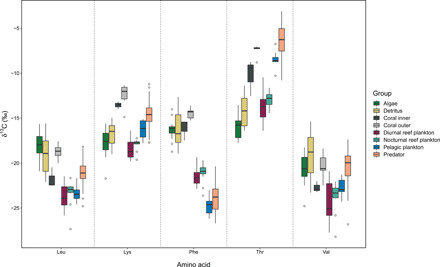
Stable isotope values of five EAAs (δ^13^C_EAA_). δ^13^C_EAA_ values of the seven specialized consumer groups sampled to represent food sources and the predators. Amino acids are as follows: leucine (Leu), lysine (Lys), phenylalanine (Phe), threonine (Thr), and valine (Val).

We measured the δ^13^C values of five EAAs [leucine (Leu), lysine (Lys), phenylalanine (Phe), threonine (Thr), and valine (Val)] from 72 samples of four species of grouper and 67 samples of eight specialized consumer species (as indicators of distinct carbon pathways) across inner and outer reef sites of an oceanic atoll. Ranges of Thr and Phe δ^13^C values were greatest (14.63 and 14.06‰, respectively), followed by Val (12.81‰), Leu (11.75‰), and Lys (10.5‰) (Fig. 2, fig. S1, and table S1).

### Some δ^13^C_EAA_ values vary spatially and among species, but not temporally

Predators were sampled twice (northeast monsoon 2017, *n* = 54; northeast monsoon 2018, *n* = 18) so we tested for temporal variation in their δ^13^C_EAA_ values. Predator δ^13^C_EAA_ values did not vary temporally, either when grouped together (year) or split by atoll habitat (year × atoll habitat) [permutational multivariate analysis of variance (PERMANOVA); table S2], so predator species from both years were combined for all analyses. δ^13^C_EAA_ values showed no spatial differences in the specialized consumer species representing algae (surgeonfish, *Acanthurus leucosternon*), detritus (surgeonfish, *Ctenochaetus striatus*), nocturnal reef plankton (soldierfish, *Myripristis violacea*), or diurnal reef plankton (fusiliers, *Caesio varilineata*; *Caesio xanthonota*) sources, so samples from both inner and outer reef habitats were pooled for each species (PERMANOVA; table S2). Furthermore, no differences were observed among diurnal reef plankton consumer species (*C. varilineata* and *C. xanthonota)* or among pelagic plankton consumer species (*Decapterus macarellus* and *Uroteuthis duvauceli*), so they were combined, respectively, into “diurnal reef plankton” and “pelagic plankton” source groups (PERMANOVA; table S2).

Peacock grouper *Cephalopholis argus* and coral hind *Cephalopholis miniata* δ^13^C_EAA_ values differed between inner and outer atolls (PERMANOVA, pseudo-*F* = 3.39, *P* = 0.04 and pseudo-*F* = 3.16, *P* = 0.05, respectively; table S2). For specialized consumers where differences in δ^13^C_EAA_ were evident, sources were kept distinct in further analysis. For the corallivorous butterflyfish, *Chaetodon meyeri*, a proxy for coral-derived carbon, δ^13^C_EAA_ values differed significantly between inner and outer atolls, so these samples were analyzed by habitat (PERMANOVA, *F*_1,7_ = 8.87, *P* = 0.03; table S2). Overall, we observed strong differences in the δ^13^C_EAA_ values among the specialized consumers representing source groups, indicating six isotopically distinct carbon pathways available to our predatory consumers (PERMANOVA, *F*_6,60_ = 30.339, *P* = 0.001). Hereafter, we refer to the specialized consumers by the source groups that they represent.

### Source groups show excellent separation using multivariate δ^13^C_EAA_ fingerprints

**Fig. 3:**
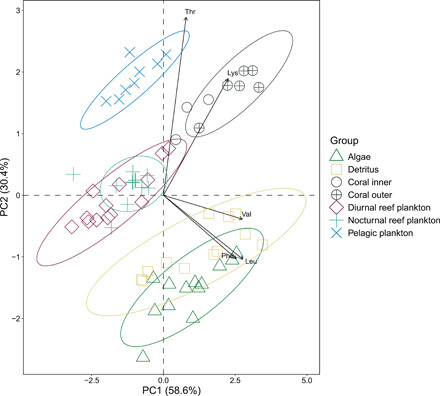
Multivariate separation of measured δ^13^C_EAA_ values of source groups. Groups displayed are the seven specialized consumer groups sampled to represent food sources, visualized using PC1 and PC2 of a PCA of the δ^13^C values of five EAA: leucine (Leu), lysine (Lys), phenylalanine (Phe), threonine (Thr), and valine (Val). Arrows show the direction and magnitude of the eigenvectors for each EAA. Dotted lines represent 95% confidence ellipses.

Principal components analysis (PCA), a multivariate technique used to visualize relationships and patterns among the δ^13^C_EAA_ values, revealed distinct separation among the source groups (Fig. 3), and the first two principal components explained 89% of the variation in the data. Separation was greater along principal component axis two (PC2 = 30.4%) that splits the groups into four distinct clusters representing the following: (i) pelagic plankton, (ii) reef-associated plankton (diurnal and nocturnal), (iii) coral, and (iv) benthic algae and detritus. The PCA loadings identified that all the amino acids, but in particular, Phe (PC1 = 0.83; PC2 = −0.34), Leu (PC1 = 0.90; PC2 = −0.34), and Val (PC1 = 0.90; PC2 = −0.13) contributed substantially to the separation of the algae and detritus from the other groups. Lys (PC1 = 0.74; PC2 = 0.62) was important in separating the coral, and Thr (PC1 = 0.26; PC2 = 0.94) was important in separating the pelagic plankton.

We achieved greater quantitative separation between the source and predator groups by comparing their multivariate δ^13^C_EAA_ fingerprints using linear discriminant analysis (LDA). LDA accounts for unique variations of δ^13^C values among the different EAAs within a single tissue type and is less rigid than Bayesian mixing models for examining diet contributions (*28*). In addition to the traditional LDA, a bootstrapping approach further maximized the source group position variability by generating “confidence zones” for each one. An initial full model of all source groups (algae, coral, detritus, diurnal reef plankton, nocturnal reef plankton, and pelagic plankton) revealed that no predators were ever classified to the algae or detritus groups (see fig. S2 and section S2 for the results of this full model). We therefore simplified the source groups into coral, reef-associated plankton (diurnal and nocturnal), and pelagic plankton, repeating the traditional and bootstrapped LDAs with these simplified groups to more finely resolve the “reef” contributions.

**Fig. 4:**
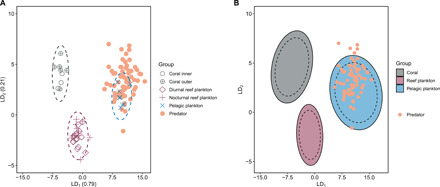
LDA of the δ^13^C_EAA_ values of the simplified source groups and predators. (**A**) LDA of the simplified source groups with predator points overlaid. Dashed lines are 95% confidence ellipses. (**B**) Confidence zones reflect the maximum area for the simplified source groups, generated from bootstrapped LDAs with 10,000 permutations and resampling. For each source group, dashed lines represent 95% confidence ellipses, while the solid lines represent 99% confidence ellipses. “Reef plankton” represents the combined diurnal and nocturnal reef-associated plankton groups. Predator points are overlaid.

There was excellent separation among source groups in the simplified model, and successful reclassification of the groups (to ensure good separation) was 100%. This confirmed that a combined reef-associated plankton group (diurnal and nocturnal) and absence of the benthic algae and detritus groups led to more accurate within-group classifications of sources and better separation of the groups along the *y* dimension LD_2_. The first linear discriminant (LD_1_) explained 79% of the data (Fig. 4A), driven by Phe and Thr. The second linear discriminant (LD_2_) explained 21% of the data, driven by Lys and Val (Table 1). The bootstrapped reclassification consistently classified the predators with the pelagic plankton group 99.98% of the time (Fig. 4B). Exceptions were individuals classified with the coral group (outer atoll *Anyperodon leucogrammicus*, 0.05%; inner atoll *C. argus*, 0.01%) or the reef-associated plankton group (outer atoll *A. leucogrammicus*, 0.03%; inner atoll *C. argus*, 1.58%; outer atoll *C. argus*, 0.01%). Overall, for both the full and the simplified LDA models, there were no differences in predator classifications spatially or interspecifically (fig. S3).

**Table 1:** Linear discriminant coefficients for the simplified LDA model. Coefficients for each EAA for LD_1_ and LD_2_ of the simplified source group (coral, reef-associated plankton, and pelagic plankton) LDA to determine which amino acids contribute to group separation.

**Amino acid**	**LD**_**1**_	**LD**_**2**_
Leucine	−0.27	0.15
Lysine	0.01	1.08
Phenylalanine	−1.48	−0.10
Threonine	1.23	0.48
Valine	−0.07	−0.67

### Predators are primarily sustained by offshore, not nearshore, production sources

**Fig. 5:**
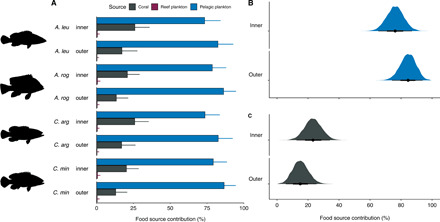
Food source contributions (%) for predators, as determined by Bayesian stable isotope mixing models using δ^13^C values of EAAs (δ^13^C_EAA_). (**A**) Food source reliance for four groupers in inner and outer atoll. Colored bars represent the mean, and thin black bars represent 95% credible intervals (CI) (2.5 to 97.5%). “Reef plankton” represents the combined diurnal and nocturnal reef-associated plankton groups. (**B** and **C**) Posterior density plots and CI of (B) pelagic plankton and (C) coral contributions to predators in the inner and outer atolls. Black dots represent the median, thicker bars represent the interquartile range (25 to 75%), and thinner bars represent the 95% CI (2.5 to 97.5%).

The LDAs allowed classification of the predators to source groups but were not able to quantify the nearshore versus offshore contributions to predator biomass. To capture the variable contributions of the three distinct pathways (nearshore: coral and reef-associated plankton versus offshore: pelagic plankton) to the different predators in both atoll habitats, a Bayesian stable isotope mixing model was run (*27*). All four groupers were primarily sustained by pelagic production in both inner [95% credible interval (CI), 64 to 89%] and outer (95% CI, 72 to 95%) atolls (Fig. 5, A and B). Median pelagic reliance was significantly greater in the outer atoll (82 to 86%) than in the inner atoll (73 to 78%) (permutation independence test, *Z* = −2.314, *P* = 0.029; Fig. 5B). Patterns in species pelagic reliance were consistent between atoll habitats: Coral hind *C. miniata* had the greatest reliance, then redmouth grouper *Aethaloperca rogaa*, peacock grouper *C. argus*, and last, slender grouper *A. leucogrammicus*. Coral-derived carbon also contributed to sustaining predator biomass (95% CI, 11 to 36% inner; 5 to 28% outer; Fig. 5, A and C). Coral median reliance was significantly higher in the inner atoll (20 to 26%) than in the outer atoll (13 to 17%) (permutation independence test, *Z* = 2.292, *P* = 0.022; Fig. 5C). Reef-associated plankton contributions were minimal in both habitats (<3%; Fig. 5A).

## DISCUSSION

Despite their oligotrophic setting, the presence and availability of planktonic inputs to coral reefs have long been recognized (*26*, *29*, *30*), but the pathways through which these subsidies are delivered, and their relative contributions, have been poorly understood. Our study adds definition to these planktonic pathways that were previously unresolved. Using δ^13^C_EAA_ fingerprints of specialized consumers, we identified two isotopically distinct planktonic pathways (nearshore reef-associated plankton and offshore pelagic plankton) and two isotopically distinct benthic pathways (coral and algae/detritus). We show that the offshore pelagic plankton pathway predominates in supporting four key abundant species of predatory reef fish across an oceanic atoll and that secondary contributions from reef-based sources are coral-derived rather than from reef-associated plankton. Our results reveal an important energetic link between shallow reefs and offshore communities, highlighting the contribution of allochthonous inputs to coral reef food webs.

A notable finding here is that the δ^13^C_EAA_ values of the local reef-associated planktivores and the offshore pelagic consumers were distinct from one another, separating nearshore reef-associated plankton from offshore pelagic plankton sources. The pelagic consumers, mackerel scad (*D. macarellus*) and Indian Ocean squid (*U. duvauceli*), are found in deeper oceanic waters, and *U. duvauceli* come to the surface to feed at night (*31*, *32*). Their δ^13^C_EAA_ values are likely a proxy for the sources sustaining the oceanic plankton community (*33*) that are distinct from those of the localized reef plankton community (*25*). Phytoplankton community composition varies between inshore and offshore reef sites (*34*), and copepod community composition and diversity is influenced by the concentration of particulate organic carbon (*35*). Given that coral reef metabolism can markedly influence seawater chemistry over short-term periods (*36*, *37*), the disparate δ^13^C_EAA_ values likely reflect differences in the dissolved inorganic carbon pools between the offshore and reef habitats. While further work is required to confirm the drivers of these different signatures, we hypothesize that the pelagic consumer δ^13^C_EAA_ values represent the offshore pelagic zooplankton advected onto coral reefs, while the δ^13^C_EAA_ values of reef planktivores represent the localized demersal reef-associated plankton pool (*25*, *26*, *38*).

The diurnal and nocturnal reef-associated plankton were also isotopically similar to each other, suggesting that the fusiliers (*C. varilineata* and *C. xanthonota*) and soldierfish (*Myripristis vittata*) could be feeding on the same local reef-based plankton supported by common phytoplankton sources (*26*, *38*, *39*). This conclusion would be unexpected as reef-associated soldierfish feed on nocturnally emerging demersal zooplankters (*39*), while highly mobile adult fusiliers feed on planktonic materials adjacent to reefs (*26*). Evidence against this hypothesis may be gained by examining the bulk sulfur isotope values (δ^34^S) of these species, which help explain movement and habitat usage (*40*, *41*). Consistent with our δ^13^C_EAA_ data, the δ^34^S values reveal a clear separation between pelagic plankton (squid, *U. duvauceli*) and reef-associated plankton (soldierfish) resources (*10*). In contrast, unlike their δ^13^C_EAA_ values that were isotopically similar, the δ^34^S values of the fusiliers (diurnal reef plankton) and the soldierfish (nocturnal reef plankton) were distinct from one another, likely reflecting that fusiliers spend more time feeding off-reef. The δ^34^S values of the fusiliers overlapped with the pelagic scad (*D. macarellus*), despite their δ^13^C_EAA_ values indicating reliance on different sources. Together, the δ^13^C_EAA_ and δ^34^S data suggest that the more mobile scad and fusiliers may feed across a reef-pelagic gradient, with the δ^34^S values reflecting their habitat usage, while the δ^13^C_EAA_ values reflect the sources most important in sustaining them and the carbon that they contribute to higher trophic levels.

The other source groups separated as expected, with benthic algae and detritus in close isotopic proximity. Although the powderblue surgeonfish (*A. leucosternon*) is an herbivore (*42*) and the lined bristletooth (*C. striatus*) a detritivore (*21*), much of the material that they are feeding on originates from what is referred to as the epilithic algal matrix (EAM), i.e., they are not strictly feeding on a single homogeneous source. Even when consumers are specialized, because of indirect trophic relationships in the food web, it is unlikely that they are sustained solely by their assumed primary food source. For example, there are small detrital contributions to the biomass of some specialized herbivores and corallivores (see section S1) (*21*, *43*). Here, the distinct separation and minimal overlap among our specialized consumers confirmed them as faithful indicators of the dominant baseline carbon sources in their diets, helping to disentangle the energetic subsidies sustaining the predators.

Offshore pelagic plankton, not nearshore reef-based sources (including reef-associated plankton), primarily sustained all predators. While recent research has highlighted this reef-pelagic linkage using stable isotopes (*7*, *8*, *10*), until now, pelagic material has been treated as isotopically homogeneous. In the Red Sea, a single pelagic plankton CSIA signature was identified (*21*); however, because of its enclosed nature and limited exchanges with the adjacent Indian Ocean (*44*), the Red Sea lacks the ready supply of offshore pelagic materials, which is a feature here. On the Great Barrier Reef, open-ocean pathways supported 57% of reef fish productivity on forereef slopes, but this was expected to be higher on oceanic reefs (*11*). Our results suggest that offshore pathways here contribute 73 to 86% to this set of high-trophic-level predators, indicative of an atoll-wide food web fueled by pelagic subsidies.

While offshore resources contributed >70% to predator diets, reef-based sources contributed 20 to 30%, and this was coral-derived rather than from reef-associated plankton. This result is unexpected given the abundant plankton-feeding fishes on these reefs (*45*, *46*), presumably reliant on the nearshore plankton pathway. Coral and reef plankton δ^13^C_EAA_ values are distinguishable from one another (*28*, *43*), supporting our specialized consumer source group separation. However, fusiliers, despite being common reef planktivores (*47*), may not entirely represent the localized reef-associated plankton in this context because of their highly mobile nature and propensity for feeding “off reef” (*48*), and it is uncertain to what degree groupers prey on them. Sampling of other more site-attached diurnal reef planktivores, such as pomacentrids (*Chromis* spp.) and serranids (*Pseudanthias* spp.), both frequently found in grouper stomach contents (*49*, *50*), is a recommended next step for this work. Moreover, when corals are more heterotrophic, their δ^13^C_EAA_ values reflect those of the zooplankton on which they are feeding (*28*, *43*). Consequently, the coral proxy here may reflect a degree of reef-associated plankton in its δ^13^C_EAA_ fingerprint. Regardless, even if the coral and reef-associated plankton source groups are considered together as “reef-based,” the offshore pelagic contribution is double that of the nearshore reef sources.

Sampling location clearly determines the degree of pelagic reliance. Maldivian reefs are highly productive, with greater mean surface chlorophyll a than Caribbean or Pacific reefs, regardless of season (*51*–*53*). Monsoonal upwelling (*54*) and equatorial currents bring allochthonous materials from further afield (*55*), while local wind-driven upwelling and internal waves facilitate linkages between deeper waters and shallow-reef communities (*53*, *56*, *57*). This enhanced productivity makes these reefs an ideal location for identifying offshore pelagic subsidies. Coral reefs in less productive regions may differ, as elsewhere consumer reliance on oceanic resources varies with proximity to the open ocean (*58*) and in relation to available primary production (*51*, *59*, *60*). In contrast, here, even inside the atoll lagoon, the predators were almost exclusively reliant on offshore production sources. Similarly, bulk stable isotope data of reef predators (*10*) and of coral host and particulate organic matter (*53*) from the region also did not differ between inner and outer reefs; extensive mixing of oceanic waters likely renders Maldivian lagoons akin to the open ocean. Our data provide further evidence of a well-mixed system where oceanic resources are abundant throughout, suggesting that, where available, higher predators in coral reef systems will access and directly benefit from open-ocean resources.

As with all emerging technologies, there is still much that is unknown about δ^13^C_EAA_ fingerprints and how accurately they transfer across trophic levels. For example, rather than being directly routed to consumer tissues from dietary sources, EAAs may be assimilated by symbiotic gut microbes (*61*) or catabolize when absorbed by gut cells (*62*). This might lead to nonzero fractionation factors between consumer and diet, affecting our ability to use indirect proxies for our sources, but is relatively unexplored (*12*). However, δ^13^C_EAA_ values of other specialized reef fish consumers align with the δ^13^C_EAA_ values of the primary carbon sources dominating their diets (*21*), confirming that they are acceptable proxies for sources. Sample analysis timing may also cause variation due to differing gas chromatograph (GC)/isotope-ratio mass spectrometer (IRMS) calibration settings (*63*). Analyses with the normalized δ^13^C_EAA_ data (see Materials and Methods and section S3), run to account for this, revealed stricter pelagic source reliance across all the predators (97 to 100%); raw data estimates were more conservative. It is therefore highly unlikely that the strong pelagic signature arises from methodological discrepancies in δ^13^C_EAA_ values. Last, although CSIA studies are increasing, very little is known about dietary incorporation rates of amino acids; there is variation among individual amino acids (*64*, *65*) and likely among taxa (*12*). However, incorporation rates of δ^13^C_EAA_ values to leopard shark muscle tissue were slow enough that dietary inferences could not be made at subannual time scales (*64*). This suggests that our predator data reflect a long-term and consistent, community-wide pattern of pelagic reliance, rather than being an artifact of sampling timing. As CSIA becomes more routine, future research should focus on how varying laboratory or GC/IRMS conditions influence δ^13^C_EAA_ values, and greater understanding of EAA integration by consumers will be required. Furthermore, it is recommended that future studies directly measure potential carbon sources where possible.

The offshore pelagic carbon subsidies that we identify here are directly fueling coral reef food webs. While coral reefs worldwide are experiencing unprecedented losses of live coral cover from persistent global bleaching events (*66*), fish productivity on those fueled by pelagic subsidies may be more resilient to coral bleaching than previously thought (*11*). Groupers, including our focal species, are a fundamental component of the Maldivian reef fishery (*67*). Their exceptionally high pelagic reliance suggests that fishery predictions based solely on habitat loss may be misleading (*68*). These results provide another compelling example of the extent of the energetic coupling between coral reefs and the surrounding ocean; these reefs are overwhelmingly reliant on external sources for maintaining their exceptional productivity. Not only do they force us to reconsider traditional views of coral reef food webs, but they also underline the importance of considering allochthonous inputs to ecosystems when studying food webs.

## MATERIALS AND METHODS

### Study locality and design

The Maldives archipelago is located in the central Indian Ocean. The north-south extent cuts across the equator and is subject to equatorial currents transporting high concentrations of nutrients (*55*). The current flow direction changes with the monsoon; during the northeast monsoon, it flows to the west, while during the southwest monsoon, it flows to the east (*54*).

North Malé Atoll (4°26′09.5″N, 73°30′01.5″E) is located in the center of the double chain of the archipelago on the eastern side. The atoll perimeter consists of an outer reef slope separated by deeper channels, while the atoll lagoon contains reef platforms (*69*). The atoll was divided into two habitats: (i) inner: lagoonal reef platform sites and (ii) outer: outer reef slope sites. Tissue sampling occurred across both inner and outer atoll habitats of North Malé Atoll (Fig. 1) during the northeast monsoon (January to March 2017 and December 2018). Mean chlorophyll a across the study region is higher during the northeast monsoon (0.32 to 1.00 mg/m^3^) than during the southwest monsoon (0.15 to 0.42 mg/m^3^) (*53*).

### Tissue sampling procedure

Groupers were chosen as representative reef predators as they are more site-attached, while other reef predators, e.g., snappers, emperors, and jacks, have larger home ranges involving long-distance movements so they may access resources from off the reef (*70*, *71*). We used results of previous survey efforts to identify groupers that dominate the abundance and biomass of the reef predator assemblage in this region (*10*, *72*). Four species were chosen that are (i) common and studied on reefs throughout the Indo-Pacific, (ii) the most abundant upper trophic level (assumed trophic level ≥ 4) groupers in both the inner and outer atoll (81% of abundance and 75% of biomass, respectively), and (iii) are key components of the local reef fishery (*67*), so the findings are relevant to sustaining the productivity of commercially valuable predators in the system. Fish were sampled using a pole spear; samples of white dorsal muscle tissue (~1-g wet mass) were removed from *A. rogaa* (redmouth), *A. leucogrammicus* (slender), *C. argus* (peacock), and *C. miniata* (coral hind). No juveniles (<15 cm) were sampled to control for ontogenetic dietary changes.

Specialized consumer species were sampled to represent distinct carbon pathways. δ^13^C_EAA_ values in consumer tissues accurately reflect the baseline carbon sources in their diet (*21*), allowing them to be used as proxies for different energy pathways. This is because producer δ^13^C_EAA_ values differ on the basis of their physiology and the biochemical processes used to shape the EAA, leading to distinct patterns in producer δ^13^C_EAA_ values, known as “^13^C fingerprints” (*19*). These patterns remain unaltered and are retained across trophic levels as the EAAs are directly routed into consumer tissues from their diet with minimal fractionation (*14*). Consumers can therefore be directly linked to their dominant dietary carbon sources with exceptional precision.

Six carbon energy pathways were identified, and specialized consumers were sampled as indicators on the basis of their carbon source reliance, accordingly: (i) benthic algae: *A. leucosternon*, powderblue surgeonfish (samples *n* = 7 inner, *n* = 6 outer) (*42*); (ii) detritus: *C. striatus*, bristletooth surgeonfish (*n* = 6 inner, *n* = 6 outer) (*21*); (iii) coral: *C. meyeri*, scrawled butterflyfish (*n* = 3 inner, *n* = 6 outer) (*73*), (iv) diurnal reef plankton: *C. varilineata*, variable-lined fusilier (*n* = 2 inner, *n* = 4 outer), and *C. xanthonota*, yellowback fusilier (*n* = 1 inner, *n* = 6 outer) (*26*, *47*, *48*); (v) nocturnal reef plankton: *M. violacea*, lattice soldierfish (*n* = 6 inner, *n* = 6 outer) (*39*); and (vi) pelagic plankton: *D. macarellus*, mackerel scad (*n* = 4 inner) (*31*) and *U. duvauceli*, Indian Ocean squid (*n* = 4 outer) (*32*). Specialized consumers were initially chosen on the basis of dietary information from the published literature and their prevalence across both atoll habitats (with the exception of the pelagic consumers). Work by the authors using bulk stable isotope data confirmed their alignment with the production sources that they represent (*10*, *74*), but assumptions about their carbon source reliance were further tested using additional δ^13^C_EAA_ data where possible (see section S1), confirming them to be faithful indicators of the primary baseline carbon sources in their diets. Samples were collected using pole spears or from a local fish market (Malé).

All tissue sampling was carried out in compliance with UK Home Office Scientific Procedures (Animals) Act Requirements. The samples were collected under research permits (OTHR)30-D/INDIV/2016/515 and (OTHR)30-D/INDIV/2018/466 granted by the Republic of Maldives Ministry of Fisheries and Agriculture, and Newcastle University Animal Welfare and Ethical Review Body approved the project (project ID: 526).

### Amino acid derivatization

Muscle tissue was oven-dried at 50°C for 48 hours and then ground to a fine powder using a pestle and mortar. *N*-acetyl isopropyl ester (NAIP) derivatives of amino acids were prepared by following the protocol described by Corr *et al.* (*75*). Briefly, this entailed hydrolysis of individual aliquots (1.5 mg) of dried powdered muscle tissue with internal standard norleucine (400 μg/ml), followed by isolation of the amino acid fraction using ion exchange chromatography with Dowex 50WX8 hydrogen form resin (200 to 400 mesh). Isopropyl esters were prepared by addition of a 4:1 mixture of isopropanol and acetyl chloride and heating for 1 hour (100°C). After removal of excess reagents by redissolving in dichloromethane (DCM) and then drying with N_2_ (40°C), acetylation was achieved by adding a mixture of acetone:triethylamine:acetic anhydride (5:2:1) and heating for 10 min (60°C). Isolation of the NAIP derivatives was achieved using liquid-liquid separation with NaCl solution (saturated) and ethyl acetate. All organic phases were combined and dried under a very gentle stream of N_2_ (room temperature). Any residual water was removed with two successive 1-ml aliquots of DCM and evaporated under a very gentle stream of N_2_ (ice bath). Samples were then stored in a freezer until they could be screened.

For screening, the derivatized amino acids were resuspended in ethyl acetate and analyzed using gas chromatography with an Agilent 7890 GC with flame ionization detection (GC/FID), fitted with a DB-35 column 30 m by 0.32 mm by 0.5 μm (Agilent), and an Agilent G4513A autosampler (Agilent Technologies, Santa Clara, CA, USA). The GC oven temperature was set to the following program: 70° (hold 2 min) to 150°C at 15°C min^−1^, then to 210°C at 2°C min^−1^, and then to 270°C at 8°C min^−1^. The injection mode was Cold on Column, and the injection volume was 1 μl with helium carrier gas at a flow rate of 2.00 ml/min.

### Stable isotope analysis

The δ^13^C isotopic compositions of the amino acids were analyzed using a GC/IRMS. A Thermo Fisher Scientific (Bremen, Germany) Delta V Plus IRMS was fitted with a Trace GC Ultra Oven, GC Isolink, and a ConFlo IV interface. The GC was fitted with a DB-35 column 30 m × 0.32 mm × 0.5 μm (Agilent). The oven was set as follows: 40° (hold 5 min) to 120°C at 15°C min^−1^, to 180°C at 3°C min^−1^, to 210°C at 1.5°C min^−1^, and then to 270°C at 5°C (hold 7 min).

Pulses of reference gas (CO_2_) were introduced into the IRMS instrument during the analysis giving rise to peaks with known δ^13^C values (^13^C:^12^C ratio relative to Pee Dee Belemnite). These reference pulses were used to calculate the analyte peaks in each chromatogram. Identification of the derivatized amino acids was achieved by matching the peak elution times with those from a mixed amino acid standard (derivatized) containing alanine (Ala), glycine (Gly), valine (Val), leucine (Leu), norleucine (Nle), threonine (Thr), serine (Ser), proline (Pro), aspartic acid (Asp), glutamic acid (Glu), hydroxyproline (Hyp), phenylalanine (Phe), lysine (Lys), and tyrosine (Tyr).

To account for the change in measured values arising from the addition of carbon atoms during the derivatization process, a correction factor was determined for each amino acid (table S3). The correction factor calculation was

$$\frac{\left((\mathrm{cd}\times \text{measured value of standard})-(c\times \text{underivatized}\;13C\;\text{value})\right)}{d}$$ (1)

where *c* is the number of carbon atoms in the amino acid, *d* is the number of carbons added during the derivatization process, and *cd* is the total number of carbon atoms in the derivative group. The correction factor for each amino acid was then applied to the raw measured values of the samples using the following equation

$$\frac{\left((\mathrm{cd}\times \text{measured value of standard})-(d\times \text{underivatized}\;13C\;\text{value})\right)}{c}$$ (2)

All samples were derivatized at Newcastle University, UK, and all GC/FID work and GC/IRMS work were carried out at the National Environmental Isotope Facility Bristol, formerly known as the Bristol Node of the NERC Life Sciences Mass Spectrometry Facility, UK. All specialized consumer source samples (except for the pelagic consumers *D. macarellus* and *U. duvauceli*) were derivatized and analyzed in 2018, while all predators and the pelagic source samples were derivatized and analyzed in 2019 (table S3).

Isotopic signatures were derived from five EAAs: leucine (Leu), lysine (Lys), phenylalanine (Phe), threonine (Thr), and valine (Val). Stable isotope ratios are reported using the delta (δ) notation with measured values expressed in per mil (‰), where δ = [(*R*_sample_ − *R*_standard_)/*R*_standard_], and *R* is the ratio of heavy to light isotope (e.g., ^13^C/^12^C).

### Statistical analysis

Analyses were carried out in R 3.6.3 (*76*) interfaced with RStudio 1.2.5042 (*77*).

### Variation in δ^13^C values of EAAs

We used PERMANOVAs [vegan (*78*), and pairwiseAdonis R packages) to investigate spatial and temporal differences in predator and source (represented by specialized consumer) δ^13^C_EAA_ values to determine where samples could be pooled. Predators were sampled during two separate years and from both inner and outer atoll, so a crossed design (year × habitat) was used. Some source groups were also sampled from both inner and outer atoll, so a one-way PERMANOVA determined whether there were spatial differences in their δ^13^C_EAA_ values. Where two species were collected to represent the same carbon pathway, a one-way PERMANOVA determined whether there were interspecific differences in their δ^13^C_EAA_ values. If there were none, then these samples were pooled into one representative source group. Last, as identifying the carbon pathways sustaining the predators relies on separation among the source groups, a one-way PERMANOVA was used to assess differences in δ^13^C_EAA_ values among the final source groups. All PERMANOVAs were run on resemblance matrices based on Euclidean distance measures and with 999 permutations.

### Multivariate separation of source groups

Relationships among the δ^13^C_EAA_ values of the source groups were initially assessed using PCA [FactoMineR R package (*79*)]. PCA is a multivariate technique used to emphasize variation and visualize patterns in a dataset, particularly when there are many variables. The PCA loadings also provide statistical estimates of the strength and direction of the effect of each variable on each principal component, identifying which amino acids drive separation among the groups.

LDA was then used to investigate the contribution of the different source groups to the predators (MASS R package (*80*)). Consumers are classified to the separated source groups using their δ^13^C_EAA_ values and underlying “fingerprints” (*19*). An initial training dataset is used to determine source classification accuracy and separation using leave-one-out cross-validation, with a high rate of reclassification of sources within their own group required to ensure that there is good separation among the sources. The training dataset is then used to predict which source group each predator will be classified to.

To further maximize the variability in the source group positions and determine whether the predators were ever classified to other source groups, we used a bootstrapping approach applied to the traditional LDA. We ran 10,000 permutations of the training dataset using random draws with replacement from each source group. For each group, ellipses were estimated for every iteration at the 95th and 99th confidence intervals around the resampled LD coordinates. Because of low sample size, coral source samples from inner and outer atolls were combined into one group. Ellipses were then drawn around these point clouds to represent confidence zones for each source group, i.e., the maximum area in which the data might fall. The predator data points were overlaid on these source groups and classified to a group.

On the basis of both the traditional and bootstrapped LDA, no predator samples were ever classified to either the algae or detritus source groups (hereafter referred to as EAM). As a result, the EAM source groups were removed from all further analysis. To more finely resolve the reef contributions, the potential reef sources were simplified into coral (benthic) or reef-associated plankton (diurnal and nocturnal). The traditional and bootstrapped LDAs were then repeated with these simplified source groups (coral, reef-associated plankton, and pelagic plankton).

### Quantifying source contributions

To capture and quantify the variable contributions of nearshore (coral and reef-associated plankton) versus offshore (pelagic plankton) sources to the different predators in both atoll habitats, a Bayesian stable isotope mixing model was run (*27*). The three source groups in the mixing model (represented by the specialized consumers and as determined with the simplified LDA) were as follows: (i) coral (*C. meyeri*), (ii) reef-associated plankton (diurnal: *C. varilineata*, *C. xanthonota*, and nocturnal: *M. violacea*), and (iii) pelagic plankton (*D. macarellus* and *U. duvauceli*). Mean and SD values were calculated for each source group to represent source means in the mixing models.

The trophic discrimination factor was set to 0.1 ± 1.0% as EAAs undergo minimal fractionation up the food chain (*21*). A larger SD value was included to provide the model with additional parameter space. Consumer data were individual grouper δ^13^C_EAA_ values. Species was included as a random factor (*A. rogaa*, *A. leucogammicus*, *C. argus*, and *C. miniata*), and habitat (inner/outer) was a fixed factor. The model was run with two error terms (process × residual), which help incorporate any variation in consumer digestibility or variation related to the sampling process (*81*). The model Markov chain Monte Carlo parameters were set to long (chain length = 300,000; burn = 200,000; thin = 100; chains = 3). Model convergence was assessed using two diagnostics: Gelman-Rubin and Geweke. The Gelman-Rubin diagnostic provides a convergence summary based on multiple chains. Model parameters with a Gelman-Rubin diagnostic of >1.1 are considered to have not converged. The Geweke diagnostic assesses convergence by comparing means from the first and last part of a Markov chain. If the samples are drawn from a stationary part of the chain, then the two means are equal, and the Geweke statistic has a standard normal distribution. Here, models were considered converged when no variables had a Gelman-Rubin diagnostic of >1.05, and based on the Geweke diagnostic, less than 5% of the variables were outside the 95% CI. Differences in the relative contribution of the dominant food sources between atoll habitats and among species were tested for using a permutation test of independence [coin R package; (*82*)].

To ensure that the timing of the sampling and of the sample analysis did not bias our interpretations, all diet contribution analyses (traditional LDA, bootstrapped LDA, and Bayesian mixing model) were carried out with the measured raw δ^13^C_EAA_ values and with values that were normalized to the sample mean (*14*). Normalizing the individual δ^13^C_EAA_ values to the mean removes potential natural variability arising from differing environmental (*14*, *22*, *23*), laboratory, or study conditions (*15*). Using this method, trends in ^13^C fingerprints are consistent, and data across studies are comparable. Here, patterns were consistent for raw and normalized data, so raw data were used throughout. The normalized data analyses, and a more detailed explanation of why the raw values were chosen, are in the Supplementary Materials (section S3).

## SUPPLEMENTARY MATERIALS

Supplementary material for this article is available at http://advances.sciencemag.org/cgi/content/full/7/8/eabf3792/DC1
